# Confounding factors in the assessment of oral mucositis in head and neck cancer

**DOI:** 10.1007/s00520-022-07128-w

**Published:** 2022-05-31

**Authors:** Luigi Lorini, Francesco Perri, Stefania Vecchio, Liliana Belgioia, Marie Vinches, Irene Brana, Sharon Elad, Paolo Bossi

**Affiliations:** 1 Medical Oncology Unit, Department of Medical & Surgical Specialties, Radiological Sciences & Public Health, University of Brescia, ASST Spedali Civili, 25123 Brescia, Italy; 2 Head and Neck Cancer Unit, Istituto Nazionale Tumori Di Napoli, IRCCS “G. Pascale”, Naples, Italy; 3 Medical Oncology, IRCCS San Martino, IST National Cancer Institute and University of Genova, Genoa, Italy; 4 Radiation Oncology Department, Health Science Department (DISSAL), IRCCS Ospedale Policlinico San Martino, University of Genoa, Genoa, Italy; 5 Montpellier Cancer Research Institute, Montpellier, Languedoc-Roussillon, France; 6 Department of Medical Oncology, Vall D’Hebron University Hospital, Vall d’Hebron Institute of Oncology (VHIO), Barcelona, Spain; 7 Oral Medicine, Eastman Institute for Oral Health, University of Rochester Medical Center, Rochester, NY USA

**Keywords:** Mucositis, Head and neck, Supportive care, Simultaneous care, Radiation toxicities

## Abstract

Treatment of locally advanced head and neck carcinoma not amenable for surgical resection or resected with high-risk features is usually based on (chemo-)radiation treatment. Oral mucositis represents one of the main side effects of (chemo-)radiation, with an important impact on quality of life and causing approximately 20% of early interruption of treatment, leading to a suboptimal dose administered. Treatment and prevention of oral mucositis have a central role in the therapeutic pathways of head and neck cancer patients but remains quite challenging. Although extensive research is conducted to identify interventions for the management of mucositis, very few interventions had sufficient evidence to generate an international expert consensus. This may be partially explained by confounding factors that could influence the development and assessment of oral mucositis. Little is known about the confounding factors of oral mucositis, which, if not well balanced in an experimental study, could lead to non-solid results. The current paper aims to review the main oral mucositis confounding factors related to head and neck cancer patients.

## Introduction

Head and neck cancer (HNC) is frequently diagnosed in locally advanced stages. Conventionally fractionated radiation therapy (RT) with concomitant chemotherapy (CT) or hyperfractionated RT combined with CT are the therapeutic standards in patients with locally advanced (LA) HNC not undergoing resection and in patients with resected high-risk LA HNC [1]. RT for HNC frequently causes oral mucositis (OM), chewing and swallowing difficulties, anorexia, xerostomia, and, consequently, weight loss. Cisplatin provides a benefit when added to RT with the cost of enhancement of treatment-related toxicities. OM is the most common side effect of RT alone or combined with chemotherapy (CRT), affecting nearly all patients (60–100%); due to OM, 20% of HNC patients discontinue RT treatment, and one out three patients therefore receive a suboptimal CT dose [2]. OM is clinically characterized by the onset of erythema, ulcers, delivering pain, eating difficulties, and, consequently, weight loss. Hence, readmission to hospital or prolongation of hospital stay is frequent due to various reasons, such as difficulty for the patient to take their domiciliary medications, the necessity to provide opioid therapy, need for total parenteral nutrition, or insert tube feeding with an important worsening in quality of life (QoL): these generate consequential economic and social costs [3, 4]. In the USA, it is estimated that the average increase in cost per HNC patient with an OM grade < 3 is up to US$1700 and for OM grade ≥ 2 is up to US$3600 from the provider perspective [2, 5].

Back to 2004, the Mucositis Group of the Multinational Association of Supportive Care in Cancer and the International Society of Oral Oncology (MASCC/ISOO) published the first clinical practice guidelines for mucositis and were reviewed periodically with the most recent update in 2020 [6]. While the guidelines are based on a systematic review, the group found very few interventions with sufficient evidence to support their use for the management of OM. These may be explained, in part, by the fact that most studies about interventions for OM are biased by confounding factors (CFs).

CFs are defined as variables that may compete with the exposure in explaining the outcome of a study. Therefore, it may mask or falsely demonstrate an apparent association. They occur when trying to determine the effect of exposure of possible risk factors on a disease; actually, the effect of another factor, a confounding variable, is measured. To be defined as a CF, the variable must satisfy three criteria: association with the disease (risk factor), association with the exposure, and not being an effect of the exposure [7]. If CFs are not well balanced in clinical trials, they may impair the quality of a study. Heterogeneity of HNC patients treated with RT or CRT exposes evaluation and assessment of OM to various CF; randomization is the way to balance CFs in the clinical trial; however, this is not always feasible in OM trials due to the small size and, frequently, the monocenter nature of the studies. CFs in OM studies could be classified into three classes: patient-related, treatment-related, and disease-related. Table 1 summarizes the main OM CFs. This paper aims to increase awareness of the importance of CFs in designing a clinical trial and in the evaluation of OM in HNC patients treated for LA disease and provide possible improvements for future trials.

**Table 1:** Mucositis confounding factors in locally advanced head and neck cancer patients treated with (chemo-)radiation

Characteristics	Effect on mucositis
***Patients-related***	
Age	Data not homogeneous
Gender	Trend for severe OM in female gender
Oral hygiene	Higher risk of OM with poor oral hygiene
Nutritional status/BMI	Higher risk of OM with low BMI
Comorbidities	Higher risk of OM with diabetes
Smoking status	Data not homogeneous
Salivatory secretory function	Higher risk of OM with low salivary secretory function
Biochemical parameters	Higher risk of OM for a low level of Hb; WBCs; lymphocyte; PLTs; and upper level of creatinine
Genetic factors	Higher risk of OM with particular genetic alterations (e.g., DNA repair genes; MDM2…)
***Disease-related***	
HPV status	Higher risk of OM in HPV-positive patients
Subsite	Higher risk of OM in the oral cavity and oropharyngeal disease
***Treatment-related***	
Radiotherapy treatment	Higher risk of OM with AF-RT and higher dose RT; proton therapy seems to reduce the risk of OM
Systemic treatment	Higher risk of OM with higher dose intensity and with cisplatin-based chemotherapy
***Others***	
Oral cavity humidification	Humidification could mitigate OM symptoms
Outcome measures	Different scale of assessment could bias the results of the OM trial

*OM*, oral mucositis; *BMI*, body mass index; *DM*, diabetes mellitus; *Hb*, hemoglobin; *WBC*, white blood cell; *PLT*, platelet; *HPV*, human papillomavirus; *AF-RT*, altered fractionated radiotherapy; RT, radiotherapy

## Patient-related confounding factors

Patient-related CFs for OM in HNC patients include age, gender, oral hygiene, nutritional status, comorbidities, smoking status, biochemical parameters, salivary secretory function, and genetic factors, as will be detailed below. However, evidence in the literature is inconsistent to support each of these CFs.

The impact of age on the development of OM was evaluated in a few studies. Merlano et al. found no statistical differences in comparing younger and older patients (age limit 65 years old) with values of grade 3 “stomatitis” (38.8% and 33.3%, respectively) and grade 4 “stomatitis” (25.4% and 32.3%, respectively) [8]. On the contrary, other series reported a higher risk of OM in younger patients [9–11]. Recently, a higher incidence and severity of OM in HNC after CRT were observed in young patients (< 45 years) when compared with older patients (> 58 years; *p* < 0.01), despite the similarities in clinical staging and treatment protocols [12]. Although data are still scarce to consider young populations at a higher risk of OM, factors, such as a higher cell renewal rate, more rapid epithelial mitotic rate, and more epidermal growth factors receptors in the epithelium of younger patients, could explain the difference in OM susceptibility [13]. On the other hand, frequent comorbidities and lower healing repair capacity could enhance OM susceptibility in geriatric patients [14]. In particular, a high prevalence of diabetes is considered a risk factor for OM [5] due to changes in microvascular gingiva and alveolar mucosa, changes in oral microbiota composition, defect of polymorphonuclear function, and abnormal metabolism of collagen [15].

Data on gender impact on OM are not homogeneous. The majority of papers reported a higher risk for OM development in female patients [16–18]. The hormonal status could play an important role; it is well known how hormonal post-translational modifications could enhance the risk of cytotoxicity and OM [19]. However, more data are needed to address this issue better; in this regard, in contrast, a few papers showed data with a higher risk of OM in male patients [20].

Wuketic et al. [21], in a prospective study on outpatients receiving CT for solid tumors, including HNC, confirmed the role of oral hygiene as CFs: the median time period since the last dental checkup was more than twice as high among the patients with clinical relevant OM [7 vs 16 months]. Poor oral hygiene influences oral microbiota and could lead to an increase in Gram-negative bacilli (GNB). The impact of oral microbiota in the development of OM after CRT for HNC patients remains debated. The presence of obligate and facultative anaerobic GNB *Bacteroidales G2*, *Capnocytophaga*, *Eikenella*, *Mycoplasma*, and *Sneathia*, as well as anaerobic GNB (*Porphyromonas* and *Tannerella*) seems to be related to grade > 2 OM. Moreover, the abundance of GNB (*Fusobacterium*, *Haemophilus*, *Tannerella*, *Porphyromonas*, and *Eikenella*) may influence the predisposal to develop OM [22, 23]. A series of 82 HNC patients treated with (C)RT [24] showed higher levels of *Cardiobacterium*; *Granulicatella*; *Prevotella*; *Fusobacterium*; *Strepotococco*; and *Megasphaere* influence the onset of severe OM. In nasopharyngeal carcinoma, RT patients harboring severe OM present a lower bacterial alpha diversity and higher abundance of *Actinobacillus* [25]. A prospective randomized trial of LA nasopharyngeal cancer undergoing CRT demonstrated that concurrent administration of probiotics led to a reduction in the rate of severe OM [26].

Nutritional status influences the development of OM; in fact, HNC patients in treatment with RT or CRT not compliant with individual dietary counseling had a greater incidence of heavy OM (88.9% vs 11.1%; *p* < 0.009) [27]. Nutritional status, evaluated with Nutritional Risk Screening (NRS) 2002 score before the start of RT and body weight loss > 5%, had been demonstrated to influence the development of OM in nasopharyngeal carcinoma patients who received CRT [28, 29]. Considering body mass index (BMI) as an indicator of nutritional status, oral cancer patients with BMI > 22 had a higher probability of developing OM during RT treatment if compared with patients with normal BMI [30–33]. Mechanisms underlying OM development in low BMI patients may derive from alterations in the host immune responses and from reduced cell migration able to repair injured tissues [31].

The relation between smoking status and OM is debated in the literature. A retrospective study showed that RT could be more effective on tumors and, at the same time, more toxic on healthy mucosa in patients without a smoking history due to higher tissue oxygenation [34]. However, other series reported a higher risk of OM in current smokers due to the pro-inflammatory activity of smoking, which could increase the damage to the mucosa [21, 35]. Heterogeneous data could be due to the fact that trials generally did not differentiate the status of smoking in “current” or “previous” and by the fact that non-smokers are usually HPV-positive [36].

Even biochemical parameters before starting CRT, such as low levels of hemoglobin, lymphocyte count, white blood cells, platelet count, and creatinine levels, upper normal limits were found to be significantly associated with the development of severe OM [20, 35, 37], as they are involved in the inflammatory response and wound healing capability.

Saliva plays a crucial role in maintaining oral mucosa and teeth health. Many factors could alter the salivary flow, for example, previous surgery or RT to the head and neck district or drugs, such as opioids. Alterations in salivary flow rate or salivary components may affect oral/mucosal health and may influence the severity of OM [38]. Low salivary flow rates at baseline and during CT were identified as risk factors for OM in a study including 63 patients receiving 5-fluorouracil as CT for a different type of cancer [10]. The composition of saliva may impact the risk for OM too: an association in the univariable analysis between an early increase of salivary cytokines (interleukin [IL]-1β, IL-6, and tumor necrosis factor-α (TNF-α)) and the development of severe OM in HNC patients treated with CRT had been found. Intriguingly, the baseline level of salivary cytokines, on the contrary, was not associated with a high risk of development of severe OM [39].

Finally, important factors linked to the increased risk of development of OM are represented by genetic alterations. It is well known that the major damage induced by RT is represented by DNA double-strand breaks, and repair pathways are of extreme importance in the resolution of radiation damage in normal tissues; an alteration, also minor as single nucleotide polymorphisms (SNPs), in genes that regulated repair of DNA damage may interfere with their function and determine the basis for increased toxicity. In the literature, trials had explained the relationship between the development of acute adverse radiation in HNC patients treated with RT and polymorphisms in DNA repair genes [40]. Available data reported a risk of grade ≥ 2 mucositis significantly increased in patients with *XRCC1*-399Gln allele genotypes both for CRT (*p* = 0.035, HR = 1.72, 95% CI = 1.03–2.86) than for RT alone (*p* = 0.049, HR = 2.50, 95% CI = 0.97–6.47) groups [41]. Interestingly, this difference seems unrelated to biologically effective radiation dose. Venkatesh et al. [42] reported that the *NBN* gene variants and haplotypes are associated with the risk of developing OM on HNC patients undergoing CT/RT. The odds of patients experiencing severe OM (grade 2) with a recessive allele of *NBN* (rs1805794) was 4.72-times higher (95% CI: 1.384–16.151; *p* = 0.013). Wardill et al. [43] recently reviewed genetic predictors of OM risk. They identified genetic factors related to pharmacogenetic variants as mutations in drug-metabolizing pathways (i.e., methylenetetrahydrofolate reductase (MTHFR), cytochrome P459, dihydropyrimidine dehydrogenase (DPYD), thymidylate synthase (TYMS), ATP-binding cassette (ABC) transporters, and glucuronosyltransferase 1A (UGT1A)); genetic factors related to cell signaling pathways (i.e., *NBN* rs 1,805,794 CC genotype, high *RPM1* gene expression, *MDM2* (309 T > G), RB1rs2227311), genetic factors related to immunogenetic variants (i.e., TNFRSR1A-610/ > G, TNFA-1211 T > C (CC genotype), and GHLR-2531C > T); and genetic factors uncategorized (*EDN1* rs1800541, *ZNF24* rs11081899-A, *APEH* c.1521G > C, and miR-1206 rs2114358). The importance of these studies is given by the need to develop a risk analysis model that could predict acute radiation effects including physical dose parameters and genotypic information. Even if a growing volume of SNP data suggests the genetic basis for susceptibility to RT-induced acute effects, it is still less clear whether the SNPs can serve as a biomarker for improved efficacy of RT. The major critic is represented by the fact that human genetic variation is very wide and we do not completely understand how this diversity could influence the phenotypic expression; this could explain the large variability in the results of available data. Moreover, it is not clear if it could be more useful to detect rare alterations having large effects or more common ones with smaller effects.

## Disease-related confounding factors

Advanced HNC may involve anatomical structures, muscles, nerves, vessels, and bones causing pain or difficulty chewing, swallowing, or moving the jaw or tongue, mucosal ulceration, and oral infections. Subsite of the primary disease plays a crucial role: incidence of OM during treatment is higher in oral and oropharyngeal tumors than hypopharynx and laryngeal tumors [44]. A retrospective analysis of 326 oropharyngeal and oral cavity squamous cell carcinoma who underwent RT with curative intent showed that patients with oropharyngeal cancer (either HPV positive or negative) had an increased risk of developing severe OM (*p* = 0.005) [20]. However, it is possible that the subsite of disease could be considered just as a proxy of total RT volume, thus influencing the risk of OM.

The impact of human papillomavirus (HPV) OM was investigated in a retrospective analysis of patients affected by oropharyngeal cancer with known HPV status treated with concurrent CT and RT [34]. HPV-positive patients had a 6.86-fold increase in the risk of having severe OM. This effect was preserved after adjusting for patient smoking status, nodal stage, RT technique, and RT maximum dose. Mechanisms of higher OM susceptibility for HPV-positive patients are unclear; maybe the same factors that contribute to the favorable response to RT (such as immune surveillance to viral-specific tumor antigens, an intact apoptotic response, absence of field cancerization) might facilitate an increased inflammatory response to (C)RT leading to a risk of OM [34].

## Treatment-related confounding factors

### Radiotherapy

Radical (C)RT is central in the treatment of LA HNC, and it is strictly related to the onset of OM. Despite technological improvement, patients developed unexpected toxicity in some cases, and it is still not clear why patients treated with the same schedule developed different toxicities. Several factors related to the onset of OM are a type of fractionation, dosimetric parameters, and RT techniques. It is known that altered fractionated (AF) RT was associated with a significant improvement in overall survival, progression-free survival, cancer mortality, local, and regional failure compared with standard fractionation radiotherapy. It is also established that AF demonstrated a higher OM incidence than the standard schedule [45]. In several randomized trials, HNC patients on AF gave the possibility to analyze the effect of different schedules on toxicity (in addition to oncological outcomes). For example, the CHART trial (54 Gy in 36 fractions over just 12 consecutive days compared to conventional schedule delivering 66 Gy in 33 fractions with five fractions per week) reported a higher incidence of confluent mucositis (75% vs 44%) and was in the CHART arm compared to the conventional one; moreover, also the peak prevalence of confluent mucositis was significantly higher (60% vs 34%) in the experimental arm with earlier onset (end of the third week vs end of the sixth week after the start of RT) [46]. Mortensen et al. [47] reported the analysis of the incidence and prevalence of acute and late morbidity observed in the 1476 patients in the DAHANCA 6 and 7 multicenter randomized trial [47]. Accelerated RT caused a significant increase in the peak incidence of OM (33% vs 53%) and the confluent OM persisted longer respect conventional group. Some data also reported the highest incidence of OM (for an accelerated schedule on 7 days per week) with a longer duration compared to the DAHANCA study (94% vs 53% with a mean duration of 4.2 vs 1.5 weeks) [48]. Moreover, with a regimen of AF-RT (radiation doses of 64.8 Gy in 3.5 weeks without CT, 1.8 Gy twice a day/5 days per week), the rate of severe OM was 84%, which was higher than observed with conventional CRT or accelerated CRT (69% and 76%, respectively) [49].

Another important factor that could confound OM registration is the wide variability in the countering of the oral cavity that could influence the reported dose and corresponding normal tissue complication probability (NTCP) model. The DAHANCA, EORTC, GORTEC, HKNPCSG, NCIC CTG, NCRI, NRG Oncology, and TROG consensus guidelines defined a surrogate structure named “extended oral cavity,” which was defined posterior to the internal arch of the mandible and maxilla [50]. This definition includes substructures not considered anatomically a part of the oral cavity; this would generate inconsistent dose–effect relationships between the dose distributions to anatomical structures involved in OM. To improve plan optimization and assure a proper interpretation and a reliable comparison of results, it is important to describe how the different structures are delineated. There has been a large effort to develop and validate accurate multifactorial NTCP models; however, the prediction of the severity of acute mucositis for individual patients is highly challenging.

The relationship between OM and dosimetric data derived from different methods of delineation (oral cavity contour, mucosal surface contours, oral cavity surface contour, oral/oropharyngeal surface contour) had been studied without finding any relation between dosimetric parameters and the duration of grade 3 OM or duration of opiate use; however, a trend towards significance between duration of strong opiate use and pretreatment weight had been shown [51]. Spatial dose distribution should be taken into account in parallel with a dose–volume histogram to predict regions where more severe OM is expected (non-keratinized vs keratinized area). Dean et al. [52] generated and validated a model to predict the severity of acute OM for individual patients and used it to establish RT dose–response associations for severe OM that could be used to inform improved RT planning.

Proton therapy is an alternative to intensity-modulated radiation therapy in treating LA HNC. Several studies showed that proton therapy significantly reduced toxicities, such as OM and dysphagia, when compared to photon-based therapy, independently by primary tumor subsites, without worsening clinical efficacy. However, currently is still unknown if clinical benefit is related to specific subsites or not [53–55].

### Systemic therapy

It is well known that CT adds survival benefit in the treatment of LA HNC than RT therapy alone, although with an increasing of toxicities. It is demonstrated that CT increases the risk of severe OM over RT alone by four times [56]. CT-induced nausea and vomiting are CFs in the evaluation of OM, potentially causing a reduction in food intake. This CF is related to the dose intensity (three weekly/weekly) and the type of CT administered, which is generally cisplatin. In 2017, a meta-analysis by Szturz et al. [57] of literature comparing weekly and three-weekly cisplatin concomitant with RT both in the post-operative and definitive setting of LA HNC. They found no difference in treatment efficacy but fewer toxicities and more compliance with weekly administration. Concerning the incidence of OM, they found a lower incidence of severe OM in the post-operative setting but not for the definitive setting. To reduce toxicities in good risk LA HNC, trials have been oriented to substituting cisplatin with less toxic agents. Analyzing the studies comparing cetuximab with cisplatin added to RT in HPV-positive cancer, the literature report that the rate of OM was not different using one or the other drug [58–60]. Similarly, the addition of avelumab to (C)RT did not cause an increase in OM in the only randomized trial [61].

### Other CFs

Humidification can play a role in the incidence of OM in patients treated with RT. Humidification can help mitigate OM symptom burden, can limit functional nutritional status decline during RT, and reduce hospitalization [62].

### Outcome measures as a source of bias

Incidence and severity of OM can be assessed with different scales: World Health Organization (WHO), Oral Mucositis Assessment Scale (OMAS), European Organization for Research and Treatment Cancer Quality of Life Questionnaire (EORTC QLQ-C30), National Cancer Institute Common Toxicity Criteria (NCI-CTC), Oral Mucositis Daily Questionnaire (OMDQ), and Functional Assessment of Cancer Therapy (FACT-G) [15]. They all have clinical and functional parameters of evaluation. Most of the scales are anatomically based and highly dependent on the training of clinicians. The WHO scale combines signs of mucosal injury with functional damage. There is no evidence about the superiority of one scale over another. However, perceptions and assessments of OM during chemoradiotherapy can differ between the patient and the physician. It could be difficult for either physician or patient to distinguish mucosal injury and symptoms related to OM due to chemo-RT or previous exposition to factors that affect mucosal status. HNC usually had previous exposure to alcohol or smoke that can cause erythroplakia, oral ulcers, mucosal fibrosis, and xerostomia; moreover, the tumor itself could invade the oral cavity or oropharyngeal structures, such as muscles, nerves, vessels, and bone causing dysphagia and pain; normal mucosal status could be damaged by production pro-inflammatory cytokines by tumors itself; finally, normal structure or oral cavity could be altered by previous surgery or RT [63].

A Patient-Reported Outcome (PRO) Assessment is a measurement based on a patient’s health status that comes directly from the patient that a doctor or any other person cannot modify or interpret. PROs should report what matters to the patient, perception of adverse events, symptoms related to the disease, and general physical condition represents an important set of information useful for improving the efficacy and tolerability of anticancer therapies. The relevant symptoms most frequently complained by HNC patients in treatment undergoing CRT are dysgeusia, pain, and OM [64]. During (C)RT for locally advanced head and neck squamous cell carcinoma, the assessment of symptoms (including OM) as evaluated by the clinicians is lower than the same assessed by PROs, particularly for low or mild symptoms (65).

### Possible solutions to overcome CF in OM studies

Based on the data mentioned above, it is clear how OM studies in HNC patients are biased by multiple CFs related to patients, disease, and treatment heterogeneity. Moreover, OM do not appear to be just a sum of CF, but a multifactorial events: in this way, large interventional studies are needed to demonstrate significant findings, where also other factors not previously considered could be taken into account and weighed in their added risk.

The first obvious way to reduce the risk of confounding factors is to conduct a randomized controlled trial that could ultimately address the diversity of included patients. Multicenter trials are needed to reach an adequate sample size and to improve the possibility to have solid data for a strong treatment recommendation for mucositis treatment, making these trials more appealing for funding. We therefore advocate that the scientific community of researchers involved in OM would join the efforts in this regard, so as to provide useable data.

Another strategy to be employed is the evaluation of new treatments for mucositis in a specific subgroup of patients, as being identified as having a higher risk of this adverse event. This could pave the way for a personalized approach according to the perceived risk. For instance, as HPV has been identified to be associated with a higher probability of developing severe mucositis, the implementation of therapeutic strategies in this subgroup of patients could be justified.

On the other side, international collaborations are eagerly awaited to support studies aimed at identifying patient, disease, and treatment characteristics that could increase the risk of mucositis. Thanks to this, it could be possible to plan future trials testing new treatments for OM prevention and/or treatment that would consider CFs as stratification factors when possible.

## Conclusion

Severe OM remains an important side effect of (C)RT of HNC, leading to patients’ worsening QoL and limiting compliance with treatment. Trials regarding OM treatment and prevention are commonly biased by confounding factors; consequently, poor data with strong efficacy evidence could help clinicians in the treatment and prevention of OM. CFs need to be prevented or removed as much as possible to understand the importance of medical interventions better. Finally, PROs instruments of assessment and physician-assessed have to be used in clinical trials. The inclusion of PROs provides information that can improve clinical management and raise the quality level of clinical trials.

## Data Availability

Not applicable.
